# The Ethics of AI Scribes as Epistemic Agents

**DOI:** 10.2196/88235

**Published:** 2026-04-30

**Authors:** Frank Ursin, Sabine Salloch

**Affiliations:** 1 Institute for Ethics, History and Philosophy of Medicine, Medizinische Hochschule Hannover, Carl-Neuberg-Strasse 1, Hannover, Lower Saxony, 30625, Germany, 49 511-532-42722, 49 511-532-165650

**Keywords:** bioethics, artificial intelligence, electronic health records, physician-patient relationship, documentation, clinical decision making, ambient documentation, epistemic agency, cognitive deskilling, narrative medicine, patient narratives

## Abstract

Artificial intelligence (AI) scribes using ambient documentation technology that capture clinician-patient dialogue and auto-generate visit notes promise to alleviate documentation burden and reduce clinician burnout. In discussing empirical evidence, highlighting research gaps, and emphasizing technology-related ethical issues beyond established AI and data ethics, we show how this promise comes along with epistemic and relational risks. We proceed in 5 steps: first, we conceptually distinguish ambient documentation from broader ambient intelligence, frame it as a “tech-fix” for documentation-related burnout, and establish the notion of AI scribes as epistemic agents rather than mere transcription tools; second, we summarize empirical evidence on AI scribes, especially with regard to their impact on physicians, highlighting risks such as cognitive deskilling, clinical deprofessionalization, and shifts in epistemic accountability; third, we analyze effects on the patient-physician relationship, focusing on relational and interpretive dimensions, including changes in communication patterns and the omission of narrative nuance; fourth, we highlight risks to patient agency and epistemic justice; and fifth, we propose a design framework for ethical deployment beyond techno-solutionism. We argue that the usefulness of AI scribes should not be justified by short-term effects, but must be assessed in the context of clinical reasoning to improve not only the working conditions of physicians, but also the quality of patient care. The paper proposes a research and design agenda to counter simple “tech-fixes” for systemic problems, envisioning AI scribes that safeguard clinical reasoning and honor patient narratives while delivering relief from documentation burdens.

## Artificial Intelligence Scribes in Clinical Care

### Clinical Documentation, Clinician Burnout, and Ethical Issues

The requirement for detailed clinical documentation imposes a heavy burden on health care professionals, manifesting both in time use and cognitive load [1]. Clinicians spend up to 50% of their shifts interacting with computers and up to 50% of that time documenting in electronic health record (EHR) systems [2], depending on the medical specialty and clinical setting [3]. In observational and survey studies, high documentation burden is strongly associated with clinician burnout, job dissatisfaction, turnover intention, and reduced time for direct patient care [4,5]. Furthermore, increased documentation demands are linked to a higher frequency of documentation errors or omissions, risks to patient safety, and lower perceived quality of care, as clinicians may rush or shortcut parts of the record [5].

Ambient documentation is envisioned to solve these problems with a combination of 2 artificial intelligence (AI)–based technologies, resulting in so-called “AI scribes” [6]. AI scribes combine ambient listening through audio recording, speech recognition, and natural language processing of the conversation during a patient visit with generative AI that extracts key clinical details from the transcript in real time, produces structured notes, drafts the documentation for the clinician to review, and then integrates it into the EHR [7-9]. Therefore, AI scribes are more than transcription tools [10,11]. In contrast to other ambient intelligence systems monitoring various practices at the patient’s bedside [12], however, AI scribes are restricted to documenting and processing oral communication and should be assessed against the common practice of physician-only documentation and the alternative of human medical scribes (Table 1).

**Table 1.** Comparison of documentation options.

	Physician	Human medical scribe	AI^a^ scribe
Presence in consultation	Physician and patient	Physician, patient, medical scribe (in-person or remote)	Physician, patient, AI scribe
Capture of conversation	Listens and records key points	Medical scribe listens and records	AI scribe records, transcribes, and processes
Note production	Writes or dictates note	Drafts note; physician edits	Generates summaries from transcripts; physician edits
Role in knowledge production	Sole epistemic agent	Recorder under supervision	Epistemic agent selects and rearranges information
Responsibility	Physician	Physician supervises scribe	Shared socio-technical process under physician’s responsibility
Scalability	Limited by physician time	Limited by staffing and costs	Highly scalable, automated, vendor-dependent

a AI: artificial intelligence.

Clinicians and nurses increasingly use AI scribe products with the intention to optimize their productivity [13]. While industry meets this demand in order to capture a promising market, the evidence on AI scribe prevalence is poor and is mostly based on surveys conducted by companies, for example, stating that nearly 40% of primary care clinicians use AI daily for clinical documentation [14]. A survey of general practitioners in Australia found that 11% have installed or tried an AI scribe, and their regular use more than doubled within 4 months, from 3% to 8% in 2024 [15]. According to the weekly online polls conducted by the Royal Australian College of General Practitioners, the percentage of general practitioners currently using AI scribes increased from 22% in 2024 to 40% in 2025 [16].

However, commentators warn that “the technology is currently too unsophisticated to improve productivity” [17]. It summarizes words spoken during a consultation but cannot replace clinical reasoning, and the time saved with the technology is lost again because clinicians need to revise AI-generated summaries and add their own inputs. In addition to these significant objections, there are concerns stemming from AI and data ethics, such as issues of data protection and privacy, bias, patient consent, hallucinations, omissions, misinterpretations, and speaker attribution errors [10,18-20]. On the one hand, there are concerns that AI scribes may lack higher levels of explicability in terms of algorithmic transparency, which poses an epistemic hurdle in identifying misinterpretations of medical terminology, leading to inaccurate documentation [21,22]. On the other hand, process transparency as the lowest level of opacity, in terms of patients not being aware that an AI scribe is used at all due to lack of disclosure, is a concern [22]. Furthermore, equity issues are raised by the observation that some clinicians avoid using AI in shared offices because of privacy worries, and patients with speech impairments might be excluded [23]. Additionally, long-term effects such as “cognitive debt” from overreliance on AI are a matter of concern [24,25].

While many studies report effects such as reduction in burnout or “pajama time,” there is a lack of ethics research examining the normative challenges of deploying AI scribes for physicians, patients, and the patient-physician relationship. For example, although loss of physician autonomy is an important topic [24], existing ethics literature currently focuses on institutional governance and justice rather than on professional autonomy [26]. Our approach will start from the concrete circumstances in clinical encounters and focus on health care providers and patients as relevant stakeholders, as well as their specific relationship in clinical consultations. We will map and describe ethical risks based on empirical evidence and ethical-epistemic concepts that are suitable for identifying the dimensions of these issues (Table 2).

**Table 2.** Ethical-epistemic dimensions of analysis of the current practice compared to new or intensified concerns regarding artificial intelligence scribes.

Ethical dimension	Current practice	New or intensified issues with AI^a^ scribes
Professional autonomy	Documentation supports reflection and memory	Automation may shift physicians into editors
Epistemic agency	Physician authors and curates the record	AI influencing knowledge formation and reliability gaps
Narrative integrity	Physician summarizes patient narratives	AI may omit narrative nuance or filter “nonclinical” content
Epistemic justice	Risk of testimonial injustice by clinicians	Epistemic failures such as speaker misattribution or errors
Situational vulnerability	Context-specific privacy and trust issues	Specialty-specific AI modes and privacy safeguards
Political economy of data	Data stored within institutional systems	Data processed by vendors and consent to (commercial) secondary uses

a AI: artificial intelligence.

Some terminological clarity is required. We will conceive AI scribes as epistemic agents, that is, entities that participate in the pursuit of epistemic goals (ie, goals in knowledge production) within clinical practice and control the formation of beliefs [27,28]. Their outputs operate at a higher level in the data-information-knowledge-wisdom hierarchy [29]: rather than merely recording or transcribing speech, they structure, select, and formulate clinically relevant information, thereby shaping the record that underpins subsequent decision-making. As reasoning increasingly unfolds within hybrid systems of human-machine interaction, AI scribes become part of a socio-technical arrangement in which data are captured, filtered, accepted, or discarded through interactions between clinicians and algorithmic tools [27]. In such settings, epistemic agency should no longer be understood as being represented solely in humans or machines; rather, it emerges relationally through the ways humans and machines jointly shape what becomes recorded as clinically relevant knowledge.

**Figure 1.**
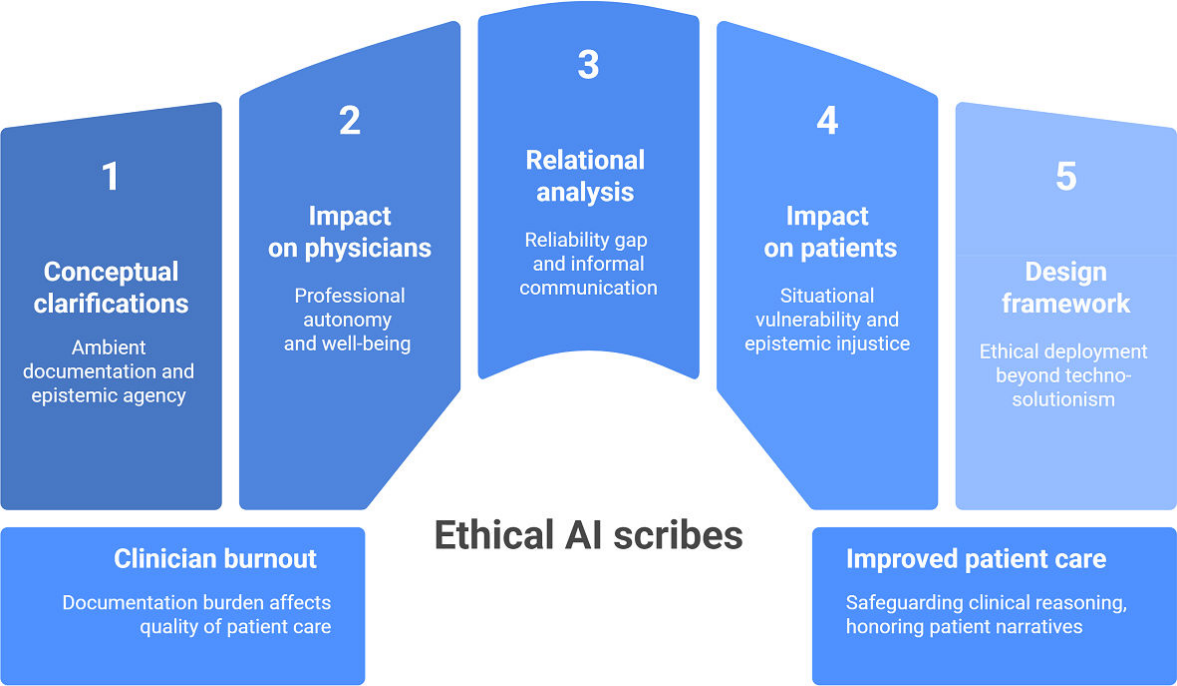
Visual abstract of steps leading to an ethical development of artificial intelligence (AI) scribes.

At the same time, AI scribes do not qualify as full artificial agents in a strong philosophical sense. According to Dung’s 5 dimensions of agency (autonomy, goal-directedness, efficacy, planning, and intentionality), current systems at least lack intentionality [30,31]. Therefore, they cannot be considered independent epistemic subjects. Nevertheless, because they perform epistemically relevant tasks, influence what becomes recorded as knowledge, and are treated as contributors to clinical reasoning, their agency is tethered to human design choices, institutional settings, and clinical use contexts [32] (p. 162). On this basis, we analyze AI scribes as epistemic agents with respect to the ethical and epistemic issues they raise for physicians, patients, and the patient-physician relationship (Table 2; Figure 1).

### Impacts of AI Scribe Use on Physician Practice

Initial evidence on how the use of AI scribes affects clinicians’ professional and personal well-being, as well as the quality of care, shows promising results, even if systematic research is still in its infancy. A survey with 1430 clinicians from 2 US centers, for example, found that ambient documentation technology was associated with reductions in burnout as well as improved well-being scores compared with baseline before ambient documentation technology use [6]. Olson et al [7] found that after 30 days with an ambient AI scribe, burnout among clinicians working in ambulatory clinics decreased from 51.9% to 38.8%. Pearlman et al [33] report from a retrospective cohort study in which clinicians who had used an AI scribe reported reductions in time spent in the electronic documentation systems compared with the control group who had not used the AI assistant. No significant differences between the 2 groups were observed, however, for after-hours time spent documenting, mean appointment length, or monthly number of completed office visits [33,34].

Even if prospective controlled research on the effects of AI scribes as health care interventions is missing so far, such initial empirical studies hint at positive consequences for physician well-being, often using the amount of after-hours or burnout indices as end points. Regarding effects on physicians, risks of deprofessionalization and loss of autonomy are highlighted by authors who see documentation as an integral part of clinical reasoning. According to McCoy et al [35], the process of taking notes, particularly in complex cases, is not “epiphenomenal” to clinical reasoning but constitutes an inherent part of it. They warn that text generated by a large language model (LLM) could reduce the overall quality of patient charts and draw a parallel: “EHR has shaped the way we think, practice, and record our patients’ stories—and so will LLMs” [35] (p. 1564). At the same time, it would be simplistic to equate typing with reasoning. The counterargument holds that reducing clerical documentation load may free cognitive resources for higher-order tasks such as diagnostic reflection and attentive listening. From this perspective, AI scribes could support rather than undermine clinical reasoning if they relieve physicians from mechanical aspects of documentation. The ethical concern, therefore, is not the delegation of typing itself but the possible erosion of epistemically productive practices such as synthesizing information, articulating provisional assessments, and identifying knowledge gaps in case of clinicians becoming passive recipients of AI-generated notes.

In addition, a “potential loss of physician autonomy” [24] is feared to be associated with the introduction of AI scribes. According to Funer and Wiesing [36], physician autonomy as an ethical principle is fundamentally anchored in the purpose of promoting a patient’s health and well-being. With the growing use of AI-based clinical decision support systems, physicians must be able to critically integrate AI outputs into their clinical reasoning to preserve professional judgment and discretion. With regard to AI scribes, the central issue is whether they retain control over how the clinical encounter is represented in the record. This requires that automatically generated transcripts and protocols remain transparent to the physician and can be revised without increasing the overall workload.

At a more foundational level, the ethical evaluation of AI scribes is intrinsically linked to the question of which purposes clinical note-taking is actually directed at. Only at first glance, AI-driven protocols can be seen as a mere documentation of what has been found, discussed, and decided in a concrete patient appointment [17]. A deeper analysis of the phenomenon needs to account for the fact that clinical notes (as all written documents) have addressees, purposes, and contexts they are serving. As indicated, AI scribes do not only (and not primarily) deliver verbatim transcripts but are designed to summarize the transcripts. Such summaries may be used not only by other health care professionals but also for insurance communication, institutional management, and statistics, and potentially by patients themselves.

Depending on the addressee and context, it is obvious that different information is needed, other parts need to be removed, and styles of language must be taken that account for the respective addressee. Due to their flexibility, LLMs might perform well to account for such different styles of reports. Ethical trade-offs, however, can occur when customizing the AI scribe for such generative functions. For example, AI-generated protocols might either serve the purposes of intraprofessional communication (and not be useful for a lay readership) or they might be appropriate for patients as readers (and thereby potentially losing medical precision). Decisions for and against certain styles for documentation, therefore, have an ethical dimension in potentially excluding certain stakeholder groups as a readership.

### AI Scribes Affecting Patient-Physician Relationship

The relationship between patient and physician is a fundamental element of health care and is even more important in times of AI-driven applications [37,38]. It is critical for ensuring effective treatment, patient satisfaction, and overall well-being. At its core, the relationship builds on trust, communication, and mutual respect, enabling physicians to provide personalized care while patients engage actively in their health management, facilitated, for example, by informed consent procedures [39]. Trust in the physician facilitates openness and encourages patients to share honest and intimate information that is needed for accurate diagnosis and effective treatment planning. At first glance, AI scribes might not influence this trustful relationship, as clinical notes usually serve the aim of professional communication. On closer look, however, automated forms of documentation can alter patient-physician communication and their relationship: “When you are being recorded, you’re going to be more careful about what you say—that’s the Hawthorne effect” [40] (p. 6).

On the one hand, first evidence shows that AI scribes strengthen the therapeutic alliance, and patients tend to trust clinicians even if they use AI scribes [41]. A consistently reported benefit is improved clinician-patient interaction during visits: clinicians focus on the patient, maintaining eye contact and active listening instead of typing on a computer [9,40,42]. An obvious advantage of using AI scribes, therefore, lies in the opportunity to better build rapport with the patient during the clinical encounter.

On the other hand, physicians’ trustworthiness can be compromised due to epistemic hurdles that prevent patients from assessing how physicians reach clinical decisions. There is a reliability gap regarding the epistemic asymmetry that arises when patients cannot clearly determine whether to trust physicians’ judgments when AI systems are involved [43]. Applied to AI scribes, this reliability gap complicates the patient-physician relationship by obscuring who accounts for and how the phrasing, selection, or omission of particular information in the medical record occurs. However, when AI scribes act as epistemic agents controlling clinical content, their output becomes part of the basis on which care is delivered. This epistemic opacity can undermine trust in clinical relationships because trust depends not only on interpersonal rapport but also on the transparency and credibility of how knowledge is formed and communicated. AI scribes reconfigure these relational dynamics.

Furthermore, there are critical issues in using AI scribes that relate to the question of which parts of the consultation are documented and which are not. Gordon Schiff [44], as a primary care practitioner, for example, emphasizes the importance of informal social conversation (“chitchat”) as an integral part of medical consultations, serving for both a fuller understanding of the patient’s health-related living situation and a sustainable patient-provider relationship. This “chitchat,” however, is deliberately omitted in AI-generated notes, not as a bug but as a feature of the system. Vendors of AI scribes often advertise their products by claiming that they only summarize “useful” information based on the SOAP (Subjective, Objective, Assessment, and Plan) categories. Schiff [44], therefore, asks whether “our society want(s) an empathetic, highly relational care delivery system built around primary care and trusting relationships” or rather develops in the direction of an efficient, convenient, highly transactional, and dehumanizing care.

It should be noted, however, that the omission of informal conversation from the formal medical record is not unique to AI-generated documentation. Clinical notes have always been selective, legally and administratively oriented documents, and social pleasantries are rarely recorded regardless of the documentation method. The ethical concern therefore does not lie primarily in the absence of such “chitchat” in the written record but in the possibility that the logic of AI-assisted documentation may gradually reshape the interaction itself in terms of communication and behavior patterns. If clinicians and patients adapt their communication styles to what is perceived as “recordable” or “clinically relevant,” informal exchanges may become less frequent even at the primary level of interaction.

### Impacts of AI Scribes on Patients

In medical encounters, clinicians often chart while patients narrate their concerns. This practice can divide clinician attention, disrupt conversational flow, and impair rapport, ultimately compromising both documentation accuracy and the quality of the patient experience [45]. Introduction of AI scribes in the clinical encounter can directly affect patients’ experiences in terms of affective states such as shame, anger, or despair, thus showing ethical significance. One immediate effect is simply a reduced human presence in the exam room, which can improve patient comfort and privacy in case of sensitive examinations [46]. By taking over the role of a medical scribe—the human alternative to AI scribes—AI-driven ambient documentation can eliminate the need for additional staff, for example, in dermatology and venereology, meaning that patients may no longer have a third party observing intimate exams [45,47].

However, it is an open question which clinical contexts are more ethically sensitive or expose more situational vulnerability [48,49]. In acute emergencies, patients may be distressed, disoriented, or unable to consent meaningfully; in psychiatric care, communication often concerns highly intimate, identity-related, or legally sensitive issues; in pediatrics, the triadic constellation between child, parents, and clinician complicates questions of privacy, assent, and representation. Deploying AI scribes identically across all these settings risks ignoring context-specific vulnerabilities and may undermine both trust and epistemic justice, for instance, if patients censor themselves because they feel surveilled or if sensitive relational nuances are systematically filtered out by the system. Design principles for AI scribes should vary by these contexts, considering pertinent issues of patient comfort and privacy as well as other aims, for example, related to security and interprofessional care.

Although we know that patients tend to judge physicians who use AI as less competent and have a significantly lower willingness to make an appointment with physicians who are using AI in general [50], there is little evidence on how patients perceive AI scribes, how much choice or control they wish to have, or how their own narratives are mediated by the AI. In an industry-initiated online survey, 57% of participants favored AI use if it reduces screen time and improves direct interaction with physicians, indicating that patients are willing to accept such technology when it enhances the human connection in care [51].

An advantage lies in the potentially increased attention physicians can now devote to capturing their patients’ illness narratives. Kleinman [52] distinguishes between *disease* (biomedical condition), *illness* (the person’s lived experience of symptoms, suffering, and changes to their identity), and *sickness* (societal meaning of being ill). By highlighting the patient’s story and the gap between physician and patient perspectives (*disease* vs *illness*), Kleinman argues that health care must attend to how people make sense of their illness, not just to how bodies malfunction. Although malfunctioning bodies are also a concern from the patient’s perspective, solely reducing health care to the professional perspective on this misrepresents holistic human experience. This aligns with the principles of narrative medicine, which emphasize the importance of honoring patients’ stories of illness as a path to better health care and empathy [53]. Narrative medicine argues that medicine practiced with “narrative competence,” that is, the ability to absorb and reflect on patient stories, leads to more humane and effective care. It is not about AI scribes recording the transcript of the patient’s illness narrative correctly but rather the positive effect of enabling doctors to practice active listening. To unlock the ethos of narrative medicine, AI scribes must help physicians listen more deeply and ensure that what enters the medical record reflects not just data but the moral and experiential truth of the patient’s story.

Capturing patient narratives is not just an esthetic or humanistic goal but can also counter epistemic injustice in health care. Epistemic injustice refers to the unfair treatment of someone as a knowledge holder [54]. In health care settings, patients have historically faced testimonial injustice, that is, their symptoms or accounts are dismissed or disbelieved, and hermeneutical injustice, that is, gaps in collective understanding that leave their experiences poorly understood due to, for example, lack of medical terminology. The framework of epistemic injustice helps to explain why, for instance, women or minority patients with pain conditions are often not taken seriously: pervasive stereotypes lead some clinicians to give their testimonies less credence. Similarly, patients with illnesses that lack clear biomedical tests (such as fibromyalgia or chronic fatigue) frequently report not having the language or credibility to explain the reality of their suffering, that is, a hermeneutical gap that leaves them feeling misunderstood.

Beyond these structural forms of epistemic injustice, AI scribes may also introduce more immediate technical biases. Speech recognition and language models often perform less accurately for speakers with strong accents, nonstandard dialects, speech impairments, or atypical prosody. In such cases, patients’ accounts may be partially misrecognized, simplified, or omitted altogether, resulting in a technologically mediated form of testimonial injustice. These disparities are not merely technical shortcomings but carry ethical significance, as they systematically reduce the epistemic credibility of certain patient groups within the clinical record.

Integrating narrative-focused AI scribes could help address such epistemic injustices by just recording the patient’s narrative to counter testimonial injustice by ensuring the patient’s account is documented in their own voice, making it harder to ignore or downplay. Likewise, broader availability of patient narratives can start to fill hermeneutical gaps that are relevant to medical research: patterns in patient-described symptoms and experiences may emerge and be analyzed, expanding the understanding of conditions that patients struggle to articulate. Hence, if AI scribes are to contribute to epistemic justice, their design should prioritize the secure preservation of verbatim patient narratives. Deleting these records, as some vendors do, risks erasing precisely the voices that narrative medicine seeks to amplify. However, the ethical appeal of preserving verbatim patient narratives must be weighed against data minimization and storage-limitation requirements as well as cybersecurity risks when retaining identifiable raw audio or transcripts. Retention of raw records can also create medicolegal exposure if a discoverable verbatim transcript diverges from the finalized clinical note. In addition, AI-generated documentation introduces the risk of “hallucinated compliance,” in which standardized sections of a note are inserted even though they were not discussed or performed. Such fabricated entries represent a distinct epistemic failure, as they can overwrite the patient’s actual testimony and create a misleading clinical record that appears complete or compliant while lacking factual grounding.

It should be emphasized that the verbatim transcript of the patient’s account follows a different structure than the SOAP framework for the purposes of physicians. From the patients’ perspective, there are also practical advantages in terms of remembering and understanding medical information when they have access to the results of the AI scribe, which are then tailored to their needs. Patients often struggle to remember what has been said during a clinical encounter: up to 80% of the medical information given by physicians is forgotten immediately, and much of what is remembered is incorrect [55]. Providing patients with a transcript or summary of the conversation can be a remedy to this catastrophe, which has significant disadvantages for the therapeutic alliance and adherence.

Indeed, clinical studies have found that patients have much better recall of information when they receive audio recordings or transcripts of their visits [56], but this is not the exact application area of AI scribes (but may be a beneficial technical design aspect). Nevertheless, by automatically generating written summaries of the consultation, an AI scribe can help patients review the doctor’s instructions, medication changes, and explanations at their own pace after the appointment. This memory aid may benefit the patient’s autonomy, therapeutic adherence, and compliance, since patients who understand and remember their options are better equipped to participate in their care decisions [17]. However, any recommendation to increase retention of transcripts must be reconciled with data minimization principles, cybersecurity risks, and liability exposure created by discoverable verbatim records.

### Ethical Design Recommendations for AI Scribes

From a critical perspective, the use of AI scribes can be conceived as a “tech-fix” [57] for systemic problems in health care systems without correcting the underlying causes in organizational structures, documentation logic, or incentive systems. A well-known feature of technical fixes is that the solution to a problem may become a problem itself [57]. However, if we are willing to accept the consequences of technological deployment, then this tech-fixing may ethically be justified through its overall net benefits. We identified ethical risks ranging from potential deterioration of clinical reasoning and physician autonomy to threats against patients’ trust in physicians, reliability gaps, and the call for narrative integrity and epistemic justice. Against this background, we outline 4 key design recommendations for AI scribe developers. Implementing these recommendations could help ensure that AI scribes alleviate clinician burnout without undermining the ethical and epistemic foundations of clinical practice.

### Mitigate Cognitive Deskilling and Support Clinical Reasoning

If physicians become passive editors of AI-produced notes, their reflective practice and case memory may atrophy over time, rendering documentation more extensive but less meaningful. Automation bias, that is, the tendency to uncritically accept AI outputs, further exacerbates the risk of deskilling by undermining independent verification and careful reasoning—at least a risk patients fear to witness [58]. Therefore, we recommend designing AI scribes as *cognitive aids* rather than replacements for clinical reasoning. AI scribes should require active clinician engagement in the documentation process to assert their abilities and competencies. For example, the AI scribe could prompt physicians to confirm or refine key assessments and conclusions rather than auto-populating them. In this scenario, clinicians are not only the human in the loop who must review and edit the AI’s note before finalizing. It rather preserves the physician’s mental models, experiences, and clinical judgment but also serves as a check against AI errors.

### Support Physician Autonomy and Epistemic Agency

Physician autonomy is a cornerstone of professional ethics, grounding the clinician’s responsibility to exercise independent judgment in the care of patients. Yet, the introduction of AI scribes may erode physician autonomy, resulting in a slippery slope of deprofessionalization: delegation of clinical reasoning to AI scribes. If AI systems assume too much control over how encounters are represented, they also impair physicians’ epistemic authority [31], that is, the competence to define what counts as relevant, true, and meaningful in the clinical record. In doing so, AI scribes risk threatening the epistemic agency of physicians through their own epistemic agency. Therefore, AI scribes must be designed to reinforce, not replace, professional judgment.

### Safeguard Patients’ Authentic Narratives

Beyond professional autonomy, AI scribes also raise epistemic concerns for patients. When AI scribes selectively capture, summarize, or “clean” patients’ illness narratives, then they may inflict epistemic injustices on patients. If AI scribes effectively become the gatekeepers of what enters the medical record, they function as epistemic agents tethered to the design choices of their developers. Therefore, AI scribes should be designed for narrative preservation under data minimization constraints. Rather than default long-term retention of raw audio or transcripts, AI scribe systems should only preserve narrative data (1) if patients have given informed consent to the processing of these data. Furthermore, (2) these data should be stored within a “contestability window” during which they can be accessed for verification and as a memory aid before default deletion. Another future challenge is the navigation of situations in which options for selective pausing or revision at the patient’s or physician’s request should be reasonably provided.

However, preserving verbatim patient narratives also raises questions about data ownership and the political economy of clinical AI systems. Many AI scribes are developed and operated by private vendors who may gain access to large-scale datasets of intimate patient-physician conversations. Without appropriate governance, the ethical goal of narrative preservation could unintentionally enable the commercial accumulation and secondary use of highly sensitive data for model training or product development. Therefore, governance should aim at purpose limitation, institutional data stewardship, consent procedures for data protection, and explicit restrictions on secondary uses of conversational data.

### Tailor AI Scribes to Clinical Settings and Situational Vulnerability

Different clinical contexts such as psychiatry, pediatrics, emergency medicine, oncology, or sexual and reproductive health expose patients to varying degrees and types of situational vulnerability [49]. Therefore, AI scribes should be designed and implemented as context-sensitive technologies, not as generic, one-size-fits-all tools. This includes enabling configurable “modes” that reflect different ethical priorities in different contexts, which must be weighed against excessive alerts or mandatory confirmations leading to workflow friction and “alert fatigue.” Complementary approaches such as periodic post hoc auditing or selective review mechanisms may provide safeguards without burdening the clinical encounter. In practice, however, encounter-level configuration may be difficult to implement in enterprise-wide EHR environments with standardized licensing and infrastructure. Context sensitivity may therefore need to be realized at different levels, for example, through institutional policies, specialty-specific templates, workflow settings, or deployment guidelines rather than through fully individualized system modes. For example, there could be default data minimization and stronger consent routines in psychiatry and sexual health; emphasis on continuity-of-care documentation in chronic care; or heightened attention to parental and surrogate roles in pediatrics. In high-vulnerability settings, the default may need to be more restrictive in terms of clearer in-room signaling when recording is active. Context-sensitive deployment also has a temporal dimension: some phases of care (eg, initial crisis intervention, breaking bad news, and end-of-life conversations) may justifiably be documented more sparsely or with delayed AI support, to protect emotional safety.

## Conclusions

AI scribes promise meaningful relief from documentation burden, yet their integration into clinical practice raises ethical and epistemic questions that reach far beyond mere workflow efficiency. Viewing these systems as epistemic agents makes visible how they participate in shaping clinical reasoning, mediating patient narratives, and influencing trust within the patient-physician relationship. Their deployment must therefore be guided by context-sensitive design principles that preserve professional autonomy, safeguard patients’ epistemic agency, and respect the situational vulnerabilities inherent in different clinical settings. Rather than functioning as simple “tech-fixes,” AI scribes should be developed and evaluated as tools that augment clinicians’ cognitive work and the relational foundations of health care. If AI scribes are designed not only to reduce documentation burden, they may also contribute to better health care.

## Abbreviations

AI: artificial intelligence
EHR: electronic health record
LLM: large language model
SOAP: Subjective, Objective, Assessment, and Plan
